# Hierarchical Porous Chitosan Sponges as Robust and Recyclable Adsorbents for Anionic Dye Adsorption

**DOI:** 10.1038/s41598-017-18302-0

**Published:** 2017-12-22

**Authors:** Mei Wang, Yifei Ma, Yan Sun, Sung Yong Hong, Stephanie K. Lee, Bumyong Yoon, Long Chen, Lijie Ci, Jae-Do Nam, Xuyuan Chen, Jonghwan Suhr

**Affiliations:** 1 State Key Laboratory of Quantum Optics and Quantum Optics Devices, Institute of Laser Spectroscopy, Collaborative Innovation Center of Extreme Optics, Shanxi University, Taiyuan, Shanxi 030006 China; 2 Department of Energy Science, Sungkyunkwan University, Suwon, 440-746 South Korea; 3 Department of Polymer Science and Engineering, Sungkyunkwan University, Suwon, 440-746 South Korea; 4 SDU & Rice Joint Center for Carbon Nanomaterials, Key Laboratory for Liquid-Solid Structural Evolution & Processing of Materials (Ministry of Education), School of Materials Science and Engineering, Shandong University, Jinan, 250061 China; 5 Department of Micro- and Nanosystem Technology, Faculty of Technology and Maritime Sciences, University College of Southeast Norway, 3184 Borre, Norway; 6 School of Mechanical Engineering, Sungkyunkwan University, Suwon, 440-746 South Korea

## Abstract

Biomass waste treatment and detrimental dye adsorption are two of the crucial environmental issues nowadays. In this study, we investigate to simultaneously resolve the aforementioned issues by synthesizing chitosan sponges as adsorbents toward rose bengal (RB) dye adsorption. Through a temperature-controlled freeze-casting process, robust and recyclable chitosan sponges are fabricated with hierarchical porosities resulted from the control of concentrations of chitosan solutions. Tested as the adsorbents for RB, to the best of our knowledge, the as-prepared chitosan sponge in this work reports the highest adsorption capacity of RB (601.5 mg/g) ever. The adsorption mechanism, isotherm, kinetics, and thermodynamics are comprehensively studied by employing statistical analysis. Importantly and desirably, the sponge type of chitosan adsorbents exceedingly facilitates the retrieving and elution of chitosan sponges for recyclable uses. Therefore, the chitosan sponge adsorbent is demonstrated to possess dramatically squeezable capability with durability for 10,000 cycles and recyclable adsorption for at least 10 cycles, which provides an efficient and economical way for both biomass treatment and water purification.

## Introduction

In our evolution towards a more sustainable society, ‘trash to treasure’ is an eternal topic of the relevant research areas seeking profits from the recycled wastes. Biomass waste is one of the reliable candidates to be recyclable, offering benefits due to its environmentally friendly and low-cost features, as well as virtually unlimited supplies. For example, chitosan, which mostly results from crustaceans including crabs, shrimp, crayfish, and lobsters, is known to be the second most abundant biopolymer in nature^1^. Worldwide, chitosan can be accumulated for 6–8 million tons every year and contain various bio-functional groups such as –NH_2_ and –OH^2^. It has been widely applied in various fields, such as artificial skin^3^, food and nutrition^4^, ophthalmology^5^, textile finishing^6^, batteries^7,8^, drug-delivery system^9^, biotechnology^10,11^, etc.

One of the important applications of chitosan is considered to be used as an adsorbent for the removal or recovery of organic/inorganic substances from aqueous solutions^12,13^. Water pollution is a vital global problem that requires persistent evaluations. The annual discharge of dyes from textile, tannery, pharmaceutical, paper, paint, plastics, petroleum, electroplating, etc. into the environment reaches about 50,000 tons, severely damaging the aesthetic nature of streams. More importantly, the coloured dyes interfere with the sunlight transmission through water, which therefore gives rise to reduction of the photosynthesis. Moreover, dyes are difficult to be biodegraded due to their complex molecular structure, in this regard, undoubtedly, removal of dyes before disposal of the wastewater is extremely important.

With the desirable features of non-toxicity, biodegradability and a high concentration of amino groups, chitosan can offer advantages in adsorbing anionic dyes from the polluted water due to the cationic nature of chitosan derived from the -NH_2_ groups. Chitosan adsorbent has been fabricated into the powder-type to enlarge the specific surface area and thus enhance the adsorption capacity, while the powder-type chitosan adsorbent suffers from the retrieving difficulties^14^. To realize the retrievability of adsorbents after the dye adsorption, magnetic materials such as Fe_3_O_4_ nanoparticles have been involved into chitosan adsorbents^15–17^. However, the complete retrieving is found to be still difficult to achieve and thus causes an unwanted secondary pollution. In addition, elution of dyes and reuse of the retrieved adsorbents still remain significant challenges for the use of the powder-type adsorbents.

In this sense, a monolith type adsorbent is preferable in the dye adsorption, which is generally in a hydrogel, foam or membrane type^18,19^, facilitating the retrievability of the adsorbent. Moreover, the porosity of the adsorbents should be controlled to pursue controllable properties such as adsorption capacity, elasticity, durability, etc. Furthermore, reliable adsorbents should possess a durable recyclability, which will prompt the use in various practical applications of the adsorbents. Chitosan chains can be easily connected with each other and form a large 3D porous scaffold. Given that the issues and essential requirements for dye adsorbents, in this study, hierarchical porous chitosan sponges as adsorbents for adsorption of rose bengal (RB), a toxic anionic dye, are fabricated by a precisely controlled freeze-casting technique. Owing to the abundant –NH_2_ groups and proper porosity of chitosan sponges, desirable adsorption performances are obtained and the adsorption mechanisms and kinetics are comprehensively studied by employing statistical analysis. At last, durable recyclability of the robust chitosan sponge adsorbents is also confirmed.

## Results and Discussion

### Hierarchical porous chitosan foams and sponges

**Figure 1:**
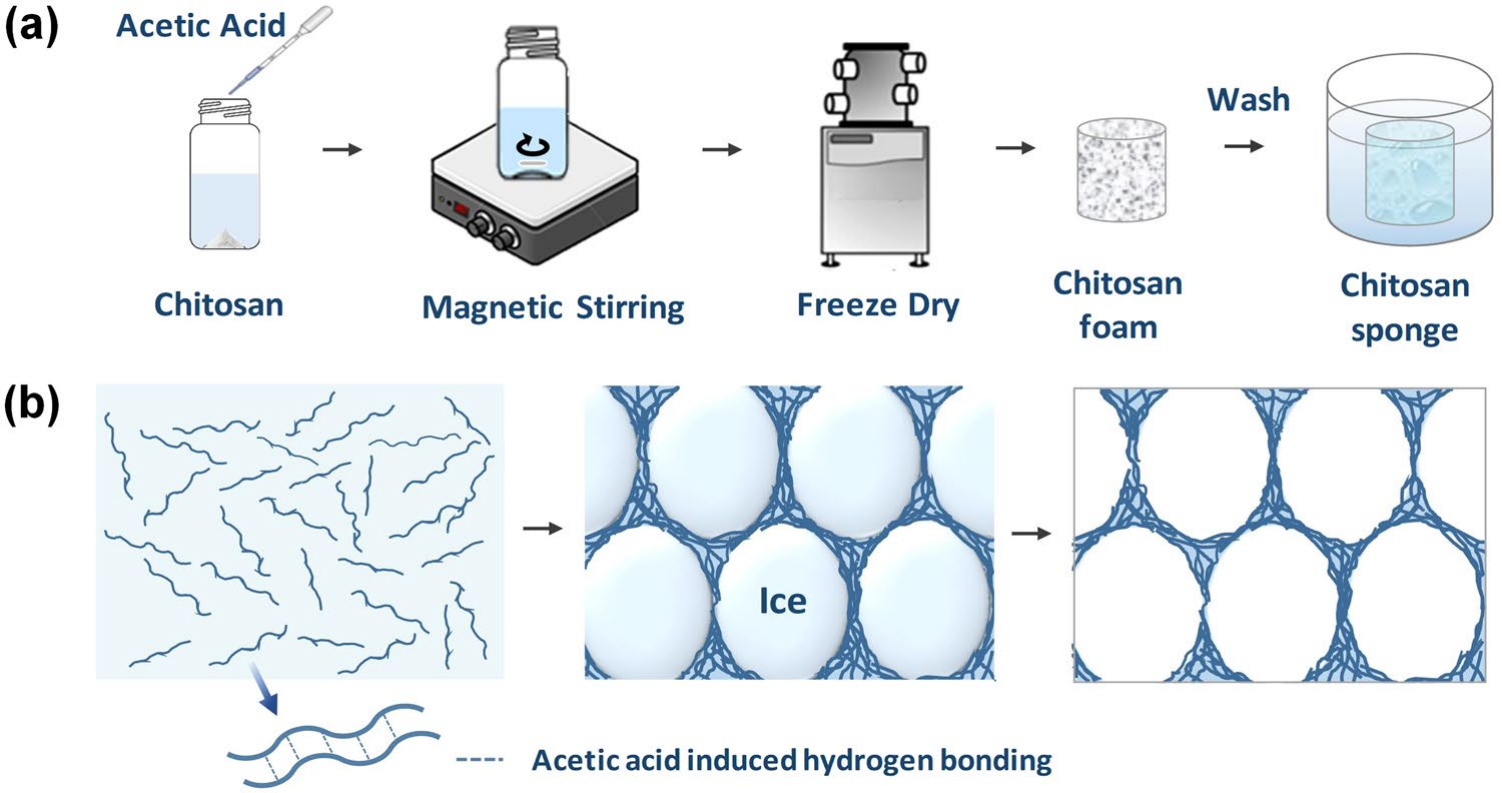
(**a**) Scheme of the synthesis process of chitosan foams and chitosan sponges. The product obtained after lyophilization is defined as chitosan foam and the washed chitosan foam saturated with water is defined as chitosan sponge. (**b**) Side-view scheme of the formation mechanism of the freeze-casting process. When the uniformly dispersed chitosan solution (left scheme) is frozen, the chitosan chains gather on the surface of the ice crystals and align along the growth direction of the ice (middle scheme). After the freeze-drying process, as a result, a chitosan foam with a continuous and uniform cellular network is formed (right scheme).

Chitosan powders cannot be dissolved in aqueous strong acid solutions, aqueous base solutions, and organic solutions because of the strong inherent hydrogen bonding. So we firstly dissolve chitosan powders into a 0.3 M acetic acid solution (Fig. 1a) where the ketonic oxygen in the carboxyl group of an acetate molecule can form a hydrogen bond with the hydroxyl group in a chitosan molecule^20^. To obtain robust and porous chitosan foams, freeze-casting method, a popular method to synthesize the foam type materials without damaging the porous structure during the drying process, is used in our study. As shown in Fig. 1b, during the freezing process, the ice crystals are formed and expanded in the solution, therefore, the chitosan chains are concentrated at the interface of the ice crystals and the solution, and aligned along the growth direction of ice crystals until the solution is fully frozen. After the subsequent lyophilization process, the chitosan can form a porous cellular structure with the chitosan chains connected with each other. The chitosan sponges are obtained after washing the residual acetic acid by a 1 M NaOH solution from the chitosan foams developed in the previous step. In this way, the density and morphology of chitosan sponges can be readily controlled by tailoring the concentration of original chitosan solutions.

**Figure 2:**
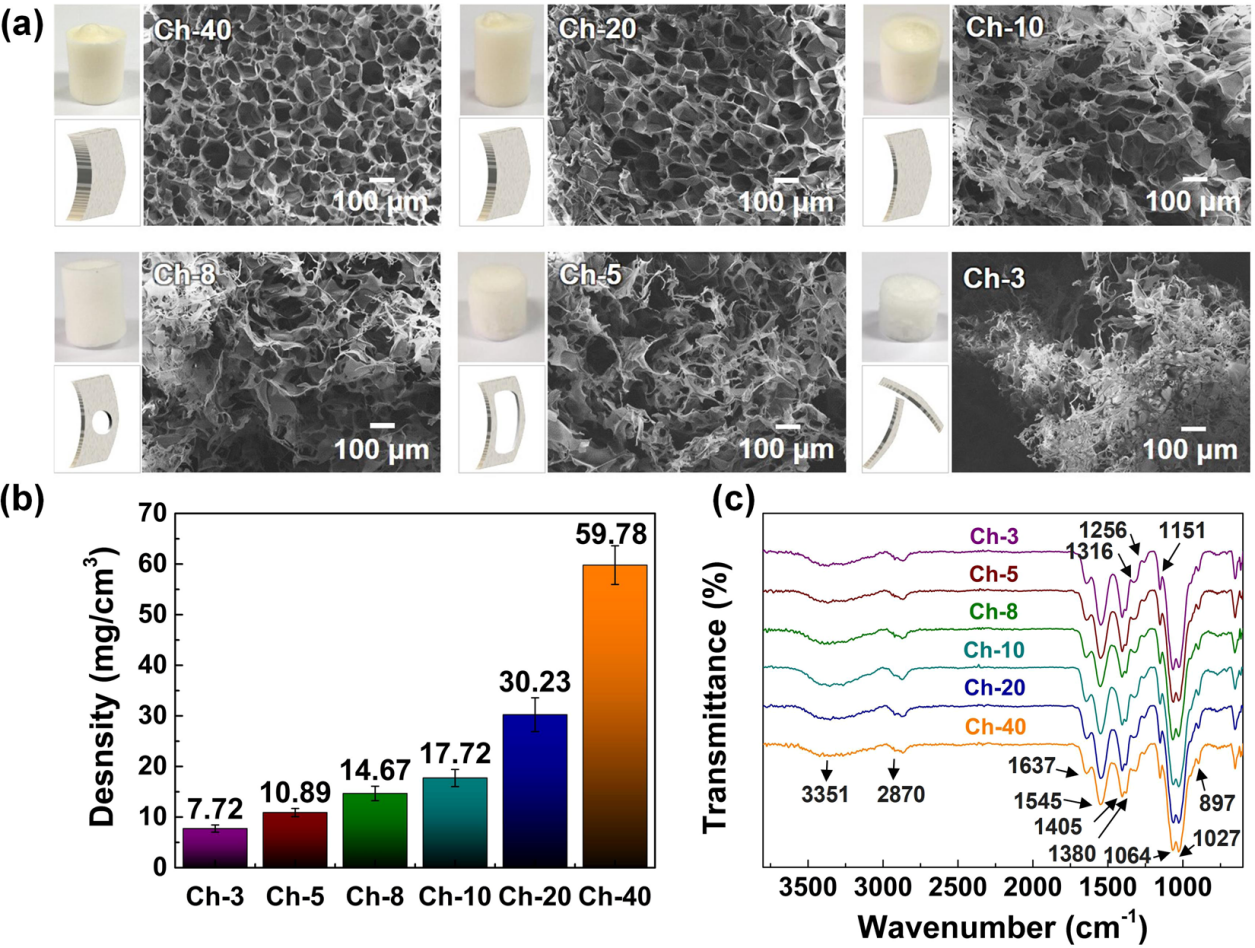
(**a**) Digital and SEM images of Ch-3, Ch-5, Ch-8, Ch-10, Ch-20, and Ch-40 chitosan foams. Schemes present the evolution process of the “cell wall” in a side view as the decrease of chitosan solution concentration. (**b**) Densities of the chitosan foams. (**c**) FT-IR spectra of Ch-3, Ch-5, Ch-8, Ch-10, Ch-20, and Ch-40 chitosan foams.

As presented in the digital pictures in Fig. 2a, all the chitosan foams are developed in a uniform cylindrical shape. The Ch-40 foam exhibits rigid while the other foams become elastic with the decrease of chitosan solution concentrations. Freezing temperature is crucial in determining the morphology of chitosan foams because the temperature difference between the atmosphere and chitosan solution plays a decisive role in the growth speed of the ice crystals. Hence, the relationship between freezing temperature and foam morphology are investigated at −20 °C, −80 °C and −196 °C. As a result, a different shape of chitosan foams is obtained at each different freezing condition (Supplementary Figure S1). Compared with freezing at −196 °C (liquid N_2_) and −80 °C, freezing at −20 °C results in the most uniform cylindrical shape of Ch-5 foams, as well as the repeatable compressibility. The chitosan foam frozen at −196 °C does not exhibit the mechanically compressible behaviour, whereas neither of the foams frozen at −80 °C nor −196 °C are developed in a desirable cylindrical shape. Here, the freezing temperature of −20 °C is determined to synthesize the chitosan foams in this study.

The hierarchical porous morphology of chitosan foams is examined and identified by the SEM images. For the foams with the high concentrations of chitosan solutions (Ch-40 and Ch-20), the chitosan foams exhibit a cellular structure with regular quasi-sphere pores and “cell walls” in the SEM images in Fig. 2a. As the decrease of the chitosan concentration, the “wall” thickness firstly becomes thinner and cellular structure cannot be formed, and subsequently pores appear on the “walls”, due to lack of chitosan chains in the solution. Finally, the “walls” turn into non-continuous and entangled “twigs”, which is confirmed in Fig. 2a. Therefore, the chitosan foams become softer, more porous and more unconsolidated, and also have lower density (Fig. 2b).

The FT-IR spectroscopy is used to identify the chemical structure of the dried chitosan foams of Ch-3, Ch-5, Ch-8, Ch-10, Ch-20, and Ch-40. As shown in Fig. 2c, the FT-IR spectra of chitosan foams with different densities indicate the identical characteristic peaks. Chitosan foams contain the peaks at 897, 1027, 1064, 1151, 1256, 1316, 1380, 1405, 1545, 1637, 2870 and 3351 cm^−1^ and the corresponding chemical associated groups are listed in the Table S1 ^21–23^. Notably, the N-containing functional groups are observed at 1256, 1316, 1545, and 3351 cm^−1^, corresponding to the stretching vibration of C-N amine II, stretching vibration of C-N amine I, bending vibration of N-H amide, and stretching vibration of N-H amide, respectively. Because of the existence of cationic amide groups, chitosan can become a good candidate as an adsorbent for anionic dyes adsorption in the polluted water.

### Chitosan sponges as RB adsorbents

**Figure 3:**
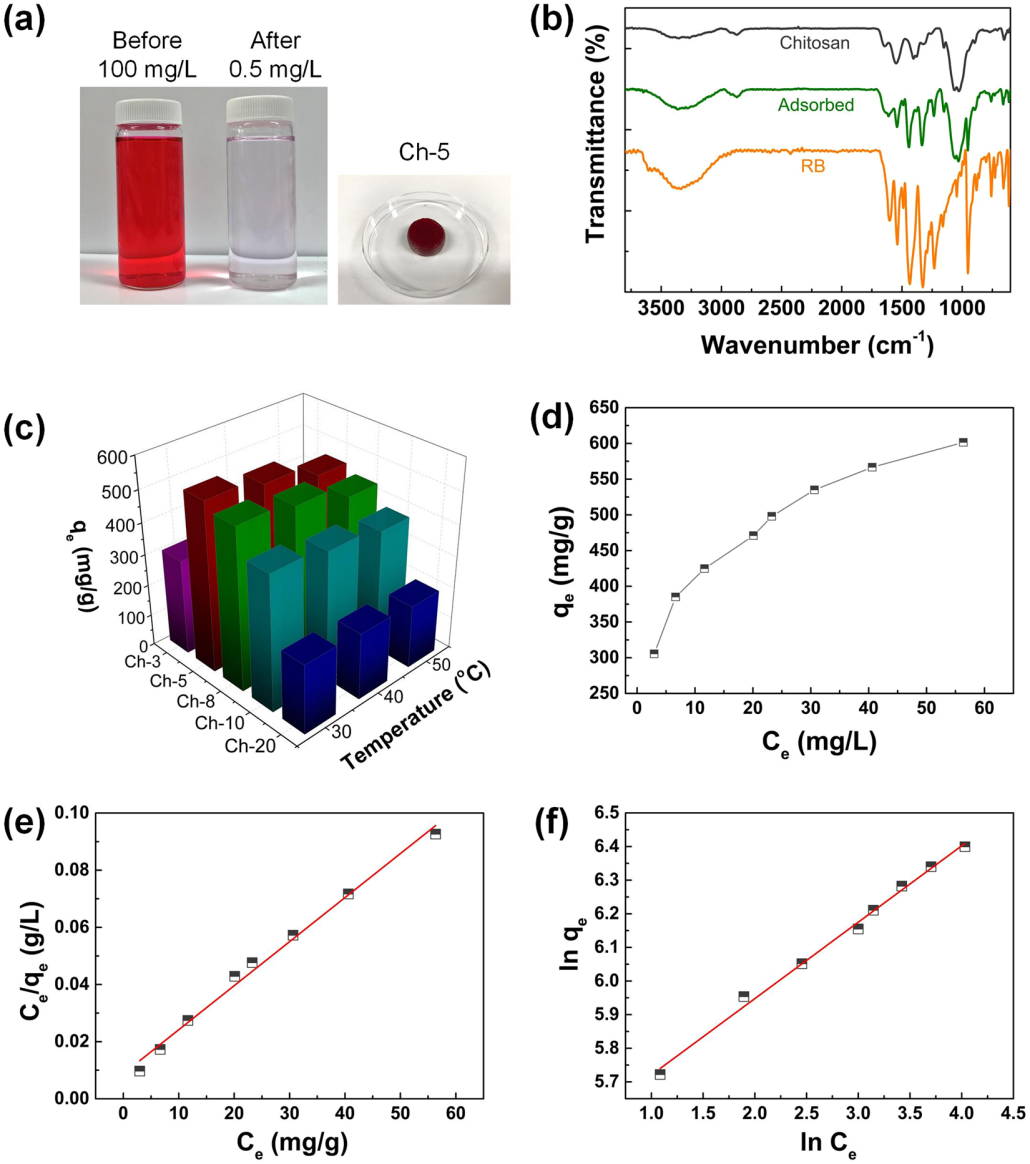
(**a**) The RB solutions before (100 mg/L) and after adsorption (0.5 mg/L) and the Ch-5 sponge after dye adsorption. (**b**) FT-IR spectra of a Ch-5 sponge, an RB adsorbed Ch-5 sponge and RB powders. (**c**) The q_e_ of chitosan sponges derived from different chitosan concentrations under different adsorption temperatures. (**d**) Adsorption isotherm of RB adsorption by Ch-5 sponges at 30 °C. (**e**) Fit of the equilibrium data with the Langmuir isotherm model. (**f**) Fit of the equilibrium data with the Freundlich isotherm model.

The as-prepared chitosan sponges were tested as the adsorbents for the anionic dye due to the cationic nature of chitosan and porous structure. Rose bengal (RB) dye is used as an example of anionic dyes to investigate the adsorption performance, mechanisms, and kinetics of chitosan sponges. A Ch-5 sponge is put into an RB solution (100 mL, 100 mg/L) and the RB is effectively adsorbed by the Ch-5 sponge (Fig. 3a). Comparing the color of the solutions before and after adsorption, barely noticeable slight pink colour remains in the solution, while the Ch-5 sponge has turned into dark red. After drying in air, the FT-IR is checked to detect the adsorption mechanism of the RB (Fig. 3b). Comparing the spectra of a RB adsorbed Ch-5 sponge, a Ch-5 sponge, and RB powders, no new peak appears for the RB adsorbed Ch-5 sponge besides the peaks existed in chitosan or RB, indicating that the adsorption is a physical process ascribed to the electrostatic interaction between anionic RB and cationic chitosan molecules.

The adsorption capacities of the Ch-3, Ch-5, Ch-8, Ch-10, and Ch-20 chitosan sponges with different densities were systematically tested under different temperatures in RB solution with the concentration of 100 mg/L (Fig. 3c and Supplementary Figure S2). Note that Ch-40 is out of selection due to the over rigidness and high density, which hinders the squeezability and RB diffusion. The adsorption capacity at equilibrium (q_e_) is calculated according to the equation as follow:

1 $${q}_{e}=\frac{({C}_{0}-{C}_{e})}{m}\times V$$

where the C_0_ (mg/L) and C_e_ (mg/L) are the concentrations of RB in the beginning and equilibrium, respectively. The C_e_ is determined by the UV-vis spectra at the length of 549 nm due to the linear relation of Abs and RB concentration (Supplementary Figure S3). m (g) is the mass of chitosan foam weighed after freeze-drying process. V (L) indicates the volume of the RB solution.

As shown in Fig. 3c, with the decrease in the density of chitosan sponges, the adsorption capacity increases because of more surface areas available for adsorption, which is confirmed by the BET specific surface area of chitosan foams (Supplementary Figure S4). However, the Ch-3 sponge does not provide the highest adsorption capability because during washing by NaOH to neutralize the residual acetic acid, some flabby parts are broken away from the sponge due to the non-continuous structure of Ch-3 foam (Fig. 2a). Therefore, the Ch-3 sponge is out of selection as a durable adsorbent due to its poor resilience. In addition, all chitosan sponges provide the best adsorption capacities at 30 °C and the highest adsorption capacity reaches 535.1 mg/g with a Ch-5 sponge (Supplementary Figure S2). Hence, the Ch-5 sponges are applied to investigate the adsorption mechanism, isotherm, kinetics, and thermodynamics, as well as the recyclability test.

### Adsorption isotherm study

In this study, Ch-5 sponges are used to investigate the adsorption isotherm at 30 °C and the capacities are plotted in Fig. 3d. The adsorption capability is dependent on the C_0_. The C_e_ is recorded at the adsorption equilibrium with different C_0_ of RB solutions (5, 10, 20, 50, 70, 100, 150, and 200 mg/L). As the increase of the C_e_, the Ch-5 sponge exhibits higher adsorption capability, which is as high as 601.5 mg/g at the C_e_ of 56.4 mg/L (C_0_ = 200 mg/L).

The adsorption isotherm describes the relationship between the amount of dye adsorbed by the adsorbent and the concentration of remaining dye in the solution. Based on the adsorption isotherm at 30 °C, the adsorption mechanism was studied using the Langmuir model and Freundlich model, which can be expressed as follows^24,25^:

2 $${\rm{L}}{\rm{a}}{\rm{n}}{\rm{g}}{\rm{m}}{\rm{u}}{\rm{i}}{\rm{r}}\,{\rm{m}}{\rm{o}}{\rm{d}}{\rm{e}}{\rm{l}}:\,\frac{{C}_{e}}{{q}_{e}}=\frac{{C}_{e}}{{q}_{max}}+\frac{1}{{q}_{max}{k}_{L}}$$

3 $${\rm{Freundlich}}\,{\rm{model}}:\,\mathrm{ln}\,{q}_{e}=\,\mathrm{ln}\,{k}_{F}+\frac{1}{n}\,\mathrm{ln}\,{C}_{e}$$

where q_max_ (mg/g), k_L_ (L/mg), k_F_ (mg/g)(L/mg), and n are the maximum adsorption capacity, the Langmuir constant, Freundlich constant, and adsorption intensity, respectively. The fitted equilibrium data with the Langmuir isotherm model is plotted in Fig. 3e. By plotting C_e_/q_e_ against C_e_, the q_max_ and k_L_ can be obtained from the intercepts and slopes^26^. The C_e_/q_e_ against C_e_ shows an approximate linear relation and the coefficient of determination (R^2^) is 0.9900. For the Freundlich model, the k_F_ and n are calculated from the plot of ln q_e_ against ln C_e_ (Fig. 3f), which also gives a good linear relation with the R^2^ of 0.9937^27^. All the equilibrium parameters obtained according to Langmuir and Freundlich models are listed in Table 1.

**Table 1:** Parameters of Langmuir and Freundlich models, R^2^, and normalized percent deviation (P) for the RB adsorption process by Ch-5 sponges under 30 °C.

Langmuir model	q_max_ (mg/g)	k_L_ (L/mg)	R^2^	P
	649.4	0.1756	0.9900	7.01
**Freundlich model**	**k**_**F**_ **(mg/g)(L/mg)**	**n**	**R** ^**2**^	**P**
	243.09	4.40	0.9937	1.21

Since the R^2^ values regarding both Langmuir model and Freundlich model are too close and equal or greater than 0.99, it is hard to determine which model should be used to better represent the RB adsorption process. The normalized percent deviation (P) is evaluated to examine the accuracy of the q_e_ collected in the experiment (Equation 4).

4 $$P=\frac{100}{N}\sum _{i=1}^{N}\frac{|{q}_{e(s)}-{q}_{e}|}{{q}_{e}}$$

The q_e(s)_ is the value of adsorption capacity simulated according to the linear fitted equation. N is the number of observations. The collected data is generally considered to be accurate when the P value is less than 5. The P value calculated in Langmuir model is 7.01, higher than 5, however, the P value calculated from Freundlich model is only 1.21. Considering the low P value in Freundlich model, we can conclude that the RB adsorption of chitosan sponges is better represented by the Freundlich model^28^. Importantly, it is noted that the calculated maximum adsorption capacity of the chitosan sponges based on the Langmuir model is 649.4 mg/g, which is higher than the capacities of RB adsorption by other reported adsorbents^15,29–34^.

### Adsorption kinetic study

**Figure 4:**
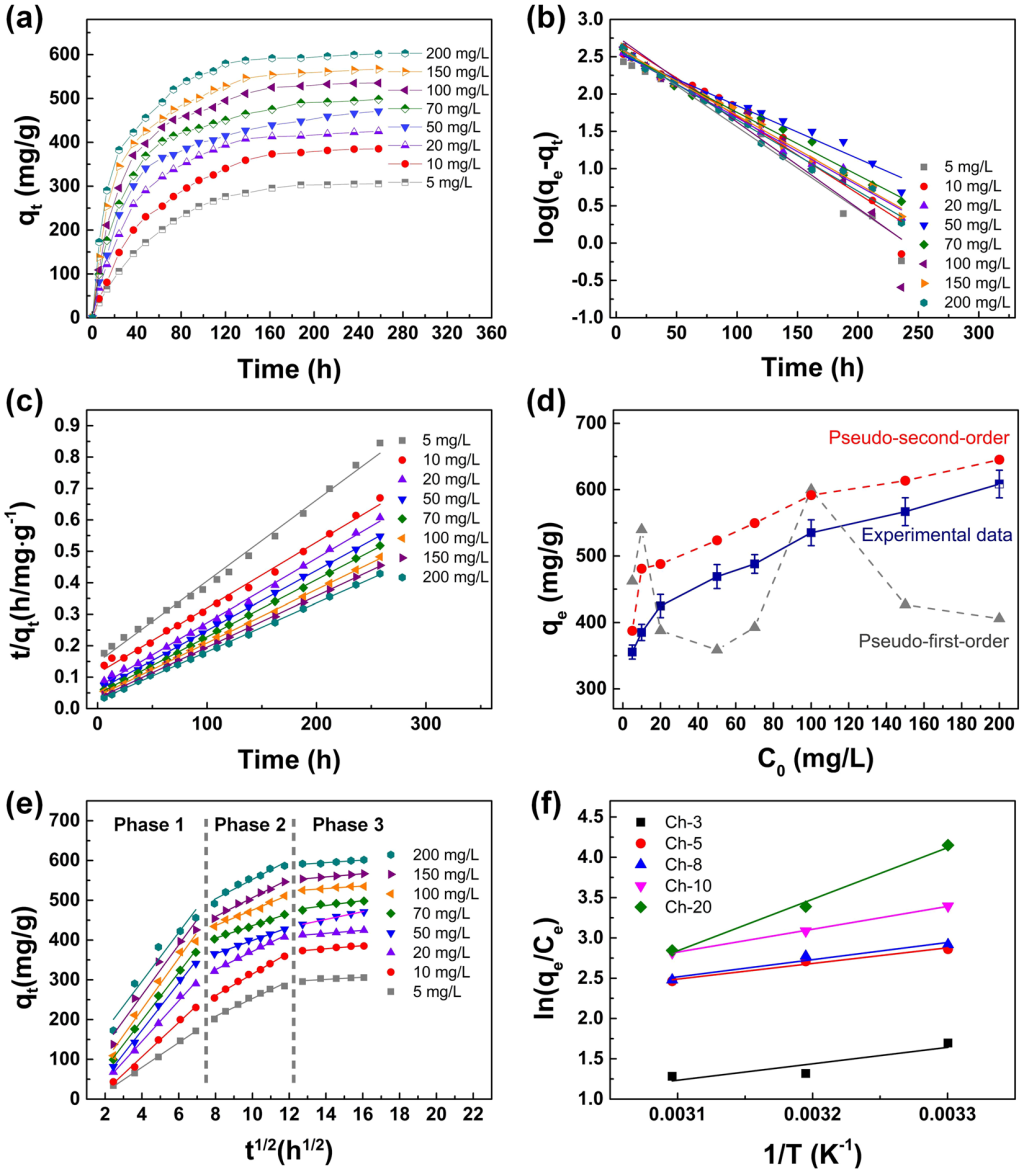
(**a**) Time-dependent adsorption of RB by Ch-5 sponges in RB solutions with different C_0_: 5, 10, 20, 50, 70, 100, 150, and 200 mg/L. The adsorption is carried out at 30 °C. (**b**) Pseudo-first-order model for the adsorption process in RB solution with different initial concentrations. (**c**) Pseudo-second-order model for the adsorption process in RB solution with different initial concentrations. (**d**) Experimental adsorption capabilities of Ch-5 sponges compared with the corresponding adsorption capacities simulated by pseudo-first-order and pseudo-second-order models in different C_0_. (**e**) Intra-particle diffusion model for the adsorption process in RB solution with different initial concentrations. (**f**) Plots of 1/T versus ln (q_e_/C_e_) for the RB adsorption at 30, 40, and 50 °C.

Adsorption kinetic models are applicable to interpret the adsorption data to gain an insight of adsorption efficiency, rate, and rate controlling step. To study the adsorption kinetics of Ch-5 sponge, the adsorption capacities against the contact time of Ch-5 sponges in RB solutions with the concentrations of 5, 10, 20, 50, 70, 100, 150, and 200 mg/L are investigated at 30 °C (Fig. 4a). Two well-known adsorption models, pseudo-first-order model^35,36^ and pseudo-second-order model^37,38^, are used to throughout investigate the adsorption mechanism and kinetics of the chitosan sponges toward RB. The equations are defined as follows:

5 $$\mathrm{Pseudo}-\mathrm{first}-\mathrm{order\; model}:\,\mathrm{log}({q}_{e}-{q}_{t})=\,\mathrm{log}\,{q}_{e}+\frac{{k}_{1}}{2.303}t$$

6 $$\mathrm{Pseudo}-\mathrm{second}-\mathrm{order\; model}:\frac{t}{{q}_{t}}=\frac{1}{{k}_{2}{q}_{e}^{2}}+\frac{t}{{q}_{e}}$$

where q_t_ (mg/g) is the adsorption capacity at a certain contact time t (h). k_1_ (1/h) and k_2_ (g/mg·h) are the rate constants of pseudo-first-order and pseudo-second-order models, respectively.

In the kinetic study (Fig. 4a), the adsorption capacity increases rapidly in the initial stage attributing to the large active surface area of Ch-5 adsorbent and limited repelling from the adsorbed RB molecules to the forwarding ones. As more RB molecules are adsorbed onto the Ch-5 sponge, the active surface area is diminished and the repelling effect becomes stronger.

The fits of the adsorption kinetic curves based on pseudo-first-order model and pseudo-second-order model are plotted in Fig. 4b and c, respectively. The values of k_1_, k_2_ and simulated q_e_ for the adsorption in RB with different C_0_ are determined from the slope and intercept of the plots (Supplementary Tables S2 and S3). The average R^2^ obtained from the pseudo-first-order model (0.9718) is much lower than the R^2^ based on pseudo-second-order model (0.9980). It indicates that the RB adsorption onto chitosan sponges is better represented by the pseudo-second-order model which refers the adsorption rate is limited by the diffusion of RB into the pores of the chitosan sponges^39^. Besides, the simulated q_e_ values by pseudo-second-order model are closer to the experimental data, compared with the capacity values by pseudo-first-order model in Fig. 4d.

However, neither the pseudo-first-order nor the pseudo-second-order models can identify the diffusion mechanism, thereby the kinetic results and the diffusion mechanism during the adsorption process are interpreted and analyzed by the intra-particle diffusion model as follow^40–42^:

7 $$\mathrm{Intra}-\mathrm{particle\; diffusion\; model}:{q}_{t}={k}_{id}{t}^{\frac{1}{2}}+{C}_{i}$$

where k_id_ (mg/g·min^1/2^) is the rate constant of intra-particle diffusion model and C_*i*_ is the intercept at stage *i*. It is obvious that three phases appear in the whole range of the plots (Fig. 4e), indicating that three stages influence the adsorption process. What’s more, the k_id_ in each step follows the order of k_1d_ > k_2d_ > k_3d_ (Supplementary Table S4), which can be ascribed to the adsorption steps of the exterior surface adsorption or instantaneous adsorption, interior surface adsorption where intra-particle diffusion is controlled, and the final equilibrium step where the solute moves slowly from larger pores to micro-pores causing a slow adsorption rate, respectively^43^. In the first stage, the adsorption rate of RB is highest due to the instantaneous availability of large active adsorption sites on the surface of chitosan sponge and the highest k_1d_ value indicates the external diffusion plays the dominant role in the adsorption kinetics^28^.

### Adsorption thermodynamic study

Tested under 30 °C, 40 °C and 50 °C (Figs 3c and 4f), the adsorption capacities of chitosan sponges decrease as the increase of temperature. This result can demonstrate the RB adsorption onto chitosan sponge is an exothermic process, coincident with the adsorption process of chitosan/graphene oxide adsorbent toward fuchsin acid dye^44^. Thermodynamic parameters, changes in the Gibbs free energy (ΔG), enthalpy (ΔH) and entropy (ΔS) during the RB adsorption process, are calculated by the following equations^28,45–47^:

8 $$\mathrm{ln}(\frac{{q}_{e}}{{C}_{e}})=-\frac{{\rm{\Delta }}H}{RT}+\frac{{\rm{\Delta }}S}{R}$$

9 $${\rm{\Delta }}G={\rm{\Delta }}H-T{\rm{\Delta }}S$$

where the T (K) is the adsorption temperature and the R (J/mol·K) is the universal gas constant. The plots of ln (q_e_/C_e_) against 1/T of Ch-3, Ch-5, Ch-8, Ch-10, and Ch-20 are presented in Fig. 4f, where the ΔH and ΔS can be calculated from the slopes and intercepts of the fits, respectively. The calculated thermodynamic data are listed in Supplementary Table S5. The negative values of ΔG suggest that the adsorption is a spontaneous process. In addition, the greater negative ΔG value indicates a more favorable adsorption, therefore, all chitosan sponges provide the highest adsorption capabilities at 30 °C over other temperatures investigated in this study. The negative values of the ΔH refers that the adsorption is an exothermic process and the negative values of the ΔS reveals the decreased randomness during the adsorption process and a less affinity of chitosan sponges and the RB molecules^27^.

### Recyclability study

**Figure 5:**
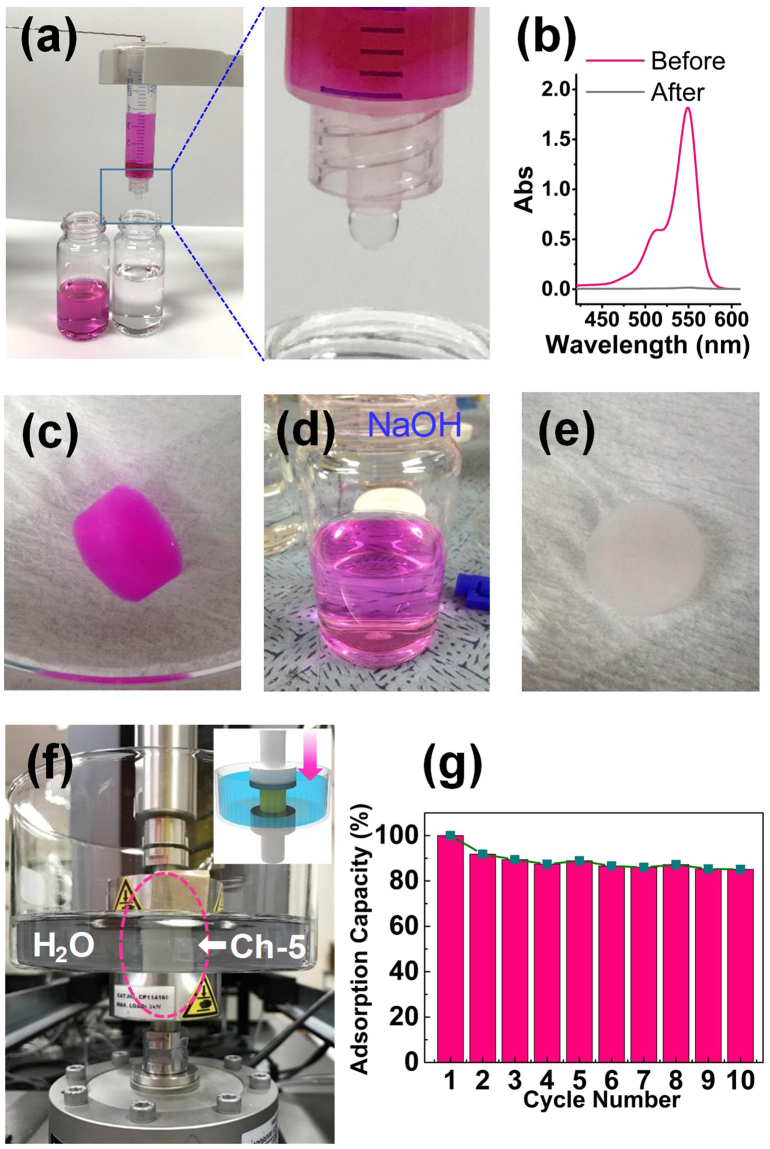
(**a**) Digital images of the apparatus for RB removal by filtration and the RB solutions before and after filtration. A Ch-5 sponge is used as the adsorbent filtration membrane. The concentration of RB used is 20 mg/L. Feeding speed of RB solution is 0.5 mL/min. (**b**) UV absorption spectra of the RB solutions before and after filtration. (**c**) The RB adsorbed Ch-5 sponge in DI-water. (**d**) A 0.5 M NaOH solution after elution process. (**e**) The Ch-5 sponge in DI-water after elution by the 0.5 M NaOH solution. (**f**) Digital image of the apparatus for compression test under water and the Ch-5 sponge after 10,000 compression cycles. The inset shows a scheme of the compression test. (**g**) Recyclability test of a Ch-5 sponge (RB concentration: 100 mg/L).

A Ch-5 sponge is loaded in a syringe in order to demonstrate the adsorption ability for filtrating the RB solution with the apparatus shown in Fig. 5a. As compared with the initial RB solution, the outlet solution becomes colourless and the concentration is only 0.12 mg/L determined from the UV absorption spectra (Fig. 5b). Desirably, the adsorbed RB on the Ch-5 sponge cannot be eluted by DI-water (Fig. 5c), indicating a strong electrostatic interaction between the chitosan and RB molecules. However, the dye on chitosan sponge can be easily eluted by squeezing the chitosan sponge under the 0.5 M NaOH solution for several seconds, while RB molecules are dissolved into the NaOH solution and the solution turns into red colour (Fig. 5d). At last, the Ch-5 sponge is washed with DI-water for several times until the pH becomes 7, and afterward, the chitosan sponge is ready for reuse (Fig. 5e).

To demonstrate the recyclability of the chitosan adsorbents, the squeezability of the chitosan sponge is tested by cyclic compressions under DI water using the apparatus in Fig. 5f. The elution process is simulated by compressing the Ch-5 sponge at a strain of up to 90% for 10,000 cycles. The height of Ch-5 sponge is completely recovered back to 100% even after 10,000 cycles of compression, demonstrating an excellent resilience and durability, as shown in Supplementary Video S1. In addition, the recyclability in terms of adsorption capability performance is also examined. A Ch-5 sponge is utilized to adsorb RB for 10 cycles and the results are presented in Fig. 5g. The adsorption capacity decreases in the second cycle noticeably and exhibits more or less constant in the subsequent cycles. After 10 cycles of adsorption, the adsorption capacity of Ch-5 sponge remains 85.1% compared with the first cycle, demonstrating the reliable recyclability of the chitosan sponge adsorbents.

Above all, the porous chitosan sponges as adsorbents offer great advantages in terms of their high controllable, recyclable, and reliable capability, which allows their hierarchal porous features, elasticity, and adsorption capacities to be tailored by varying the concentrations of the original chitosan solutions. Moreover, according to the aforementioned characterizations and analysis of chitosan sponges, we compare the adsorption performances of the Ch-5 sponge with other reported adsorbents for anionic dye adsorption, as summarized in Supplementary Table S6. These results can indicate that the chitosan sponge investigated in this study shows unprecedented outstanding capability over other RB adsorbents reported so far, not only in the adsorption capacity but also in the recyclability with reliability. Even comparing with other chitosan-based adsorbents, our chitosan sponge is also found to be comparable upon anionic dye adsorption (Supplementary Table S6).

## Conclusions

In this study, we fabricated hierarchical porous chitosan sponges by a temperature-controlled freeze-casting method, with which the morphology and porosity of the chitosan sponges can be controlled by the freezing temperature and the concentrations of original chitosan solutions. The chitosan sponges with different densities are tested as the RB adsorbents, as a result, the Ch-5 sponge exhibits the best adsorption capability (601.5 mg/g), which is the highest among the reported capacities of RB adsorbents with the best of our knowledge. The study of the responsible adsorption mechanism reveals that the adsorption is shown to be ascribed to the electrostatic interaction between RB and chitosan molecules. In addition, the investigation of the adsorption isotherm and kinetics indicates that the adsorption process is better represented by the Freundlich model for isotherm and pseudo-second-order model for kinetics, which refers that the diffusion of RB molecules into the chitosan sponges is the decisive factor to the adsorption rate. Furthermore, the thermodynamic study confirms that the adsorption is a spontaneous and exothermic process. Finally, the recyclability and durability of the chitosan sponge adsorbents are examined and verified. In conclusion, the aforementioned excellent performances of chitosan sponge can show a great promise for the use as dye adsorbents for wastewater purifications. We believe that this work could significantly contribute to our society that is rapidly facing the environmental issues, by opening up a new solution with the utilization of environmentally friendly biomass materials.

## Methods

### Preparation of chitosan sponge adsorbents

Firstly, chitosan powders were added into a 0.3 M acetic acid solution at the chitosan concentrations of 3, 5, 8, 10, 20 and 40 mg/mL, respectively. Subsequently, the solution was heated at 50 °C and stirred with a magnetic bar until the chitosan powders were totally dissolved. Then a temperature-controlled freeze-casting method was used to fabricate the porous chitosan foams. The chitosan solutions were put into 10 mL vials and frozen at −20 °C, −80 °C and −196 °C, respectively. Freezing at −20 °C and −80 °C was then carried out with refrigerator and the freezing temperature of −196 °C was achieved with liquid nitrogen. Finally, the lyophilization was carried out in a freeze-dryer at −80 °C for 48 hours to obtain the chitosan foams. The resultant chitosan foams were weighed and the densities of the chitosan foams were determined.

After the chitosan foams were dried, a certain amount of acetic acid still existed in the chitosan foam. A 1 M NaOH solution was used to wash out the residual acetic acid and then the chitosan sponge was washed in a DI-water bath with repetitively squeezing the sponge until the pH of the DI-water bath becomes 7. The water in chitosan sponge was then squeezed out and the sponge was put into the rose bengal solution for adsorption test. All the adsorption processes were carried out under shaking with a rate of 150 rpm using a temperature-controlling shaking incubator. Chitosan foams and sponges prepared in the concentrations of 3, 5, 8, 10, 20 and 40 mg/mL are named as Ch-3, Ch-5, Ch-8, Ch-10, Ch-20 and Ch-40, respectively. The chitosan sponges used in the adsorption processes were derived from 2.5 mL of chitosan solutions.

### Characterizations

The morphology of chitosan foams was characterized by a field emission scanning electron microscope (FE-SEM, JSM-7600F, JEOL). The FT-IR spectra of chitosan foams were investigated using an attenuated total reflection infrared spectroscopy (Tensor 27, Bruker) conducted at a resolution of 4 cm^−1^ with 50 scans. The Brunauer-Emmett-Teller (BET) specific surface areas of chitosan foams were measured from the N_2_ adsorption isotherm at 77 K by using the Quadrasorb SI-MP instrument. The chitosan foams were outgassed at 373 K for 12 h before BET measurement. The ultraviolet visible (UV-vis) absorption spectra were recorded using a UV–visible spectrometer (V-650, JASCO, Japan). The compression test of chitosan sponge is undertaken with a uniaxial test machine (UTM, ElectroPuls E3000 Testing System, Instron) for 10,000 cycles at a frequency of 0.1 Hz.

## Electronic supplementary material

Supplementary video S1

Supplementary Information

## References

[1] Xie SL (2015). Chitosan Waste-Derived Co and N Co-doped Carbon Electrocatalyst for Efficient Oxygen Reduction Reaction. Chemelectrochem.

[2] Huang, J., Zhong, Y., Zhang, L. & Cai, J. Extremely Strong and Transparent Chitin Films: A High-Efficiency, Energy-Saving, and “Green” Route Using an Aqueous KOH/Urea Solution. *Adv. Funct. Mater.* **27**, 1701100, 10.1002/adfm.201701100 (2017).

[3] Liu YH, Zhu LQ, Feng P, Shi Y, Wan Q (2015). Freestanding Artificial Synapses Based on Laterally Proton-Coupled Transistors on Chitosan Membranes. Adv. Mater..

[4] Chantarasataporn P (2014). Water-based oligochitosan and nanowhisker chitosan as potential food preservatives for shelf-life extension of minced pork. Food Chem..

[5] Hermans K (2014). Development and characterization of mucoadhesive chitosan films for ophthalmic delivery of cyclosporine A. Int. J. Pharm..

[6] Busila M, Musat V, Textor T, Mahltig B (2015). Synthesis and characterization of antimicrobial textile finishing based on Ag:ZnO nanoparticles/chitosan biocomposites. Rsc Adv.

[7] Yue L, Zhang LZ, Zhong HX (2014). Carboxymethyl chitosan: A new water soluble binder for Si anode of Li-ion batteries. J. Power Sources.

[8] Leones R, Sabadini RC, Esperanca JMSS, Pawlicka A, Silva MM (2017). Effect of storage time on the ionic conductivity of chitosan-solid polymer electrolytes incorporating cyano-based ionic liquids. Electrochim. Acta.

[9] Hu L, Sun Y, Wu Y (2013). Advances in chitosan-based drug delivery vehicles. Nanoscale.

[10] Ohlknecht C (2017). Cellobiose dehydrogenase and chitosan-based lysozyme responsive materials for antimicrobial wound treatment. Biotechnol. Bioeng..

[11] Zhao Y (2016). Underwater superoleophobic membrane with enhanced oil-water separation, antimicrobial, and antifouling activities. Adv. Mater. Interfaces.

[12] Vakili M (2014). Application of chitosan and its derivatives as adsorbents for dye removal from water and wastewater: a review. Carbohydr. Polym..

[13] Shen Y, Fang Q, Chen B (2015). Environmental applications of three-dimensional graphene-based macrostructures: adsorption, transformation, and detection. Environ. Sci. Technol..

[14] Ouyang A (2015). Highly Porous Core-Shell Structured Graphene-Chitosan Beads. ACS Appl Mater Interfaces.

[15] Salam MA, El-Shishtawy RM, Obaid AY (2014). Synthesis of magnetic multi-walled carbon nanotubes/magnetite/chitin magnetic nanocomposite for the removal of Rose Bengal from real and model solution. J Ind Eng Chem.

[16] Gul K (2016). Functionalization of magnetic chitosan with graphene oxide for removal of cationic and anionic dyes from aqueous solution. Carbohydr. Polym..

[17] Kim HR, Jang JW, Park JW (2016). Carboxymethyl chitosan-modified magnetic-cored dendrimer as an amphoteric adsorbent. J. Hazard. Mater..

[18] Chen YQ, Chen LB, Bai H, Li L (2013). Graphene oxide-chitosan composite hydrogels as broad-spectrum adsorbents for water purification. J Mater Chem A.

[19] Yan H, Yang H, Li AM, Cheng RS (2016). pH-tunable surface charge of chitosan/graphene oxide composite adsorbent for efficient removal of multiple pollutants from water. Chem. Eng. J..

[20] Hao P (2015). Graphene-based nitrogen self-doped hierarchical porous carbon aerogels derived from chitosan for high performance supercapacitors. Nano Energy.

[21] Pereira VA, de Arruda INQ, Stefani R (2015). Active chitosan/PVA films with anthocyanins from Brassica oleraceae (Red Cabbage) as Time-Temperature Indicators for application in intelligent food packaging. Food Hydrocolloid.

[22] Mansur HS, Mansur AAP, Curti E, De Almeida MV (2013). Functionalized-chitosan/quantum dot nano-hybrids for nanomedicine applications: towards biolabeling and biosorbing phosphate metabolites. J Mater Chem B.

[23] Smith, B. C. *Infrared spectral interpretation: a systematic approach*. (CRC press, 1998).

[24] Langmuir I (1918). The adsorption of gases on plane surfaces of glass, mica and platinum. J Am Chem Soc.

[25] Freundlich H (1906). Over the adsorption in solution. J. Phys. Chem.

[26] Du QC (2016). A graphene-melamine-sponge for efficient and recyclable dye adsorption. Rsc Adv.

[27] Duman O, Ayranci E (2010). Attachment of benzo-crown ethers onto activated carbon cloth to enhance the removal of chromium, cobalt and nickel ions from aqueous solutions by adsorption. J. Hazard. Mater..

[28] Chen L (2017). High performance agar/graphene oxide composite aerogel for methylene blue removal. Carbohydr. Polym..

[29] Gupta VK, Mittal A, Jhare D, Mittal J (2012). Batch and bulk removal of hazardous colouring agent Rose Bengal by adsorption techniques using bottom ash as adsorbent. Rsc Adv.

[30] Wu T (2013). Three-dimensional graphene-based aerogels prepared by a self-assembly process and its excellent catalytic and absorbing performance. J Mater Chem A.

[31] Wang ZX, Guo J, Ma J, Shao L (2015). Highly regenerable alkali-resistant magnetic nanoparticles inspired by mussels for rapid selective dye removal offer high-efficiency environmental remediation. J Mater Chem A.

[32] Fan JC, Shi ZX, Lian M, Li H, Yin J (2013). Mechanically strong graphene oxide/sodium alginate/polyacrylamide nanocomposite hydrogel with improved dye adsorption capacity. J Mater Chem A.

[33] Ji KJ, Xu HJ, Ma XD, Yin J, Jiang XS (2017). Hyperbranched poly(ether amine)@poly(vinylidene fluoride) (hPEA@PVDF) porous membranes for selective adsorption and molecular filtration of hydrophilic dyes. J Mater Chem A.

[34] Naushad M, ALOthman ZA, Awual MR, Alfadul SM, Ahamad T (2016). Adsorption of rose Bengal dye from aqueous solution by amberlite Ira-938 resin: kinetics, isotherms, and thermodynamic studies. Desalin Water Treat.

[35] Doğan M, Alkan M, Demirbaş Ö, Özdemir Y, Özmetin C (2006). Adsorption kinetics of maxilon blue GRL onto sepiolite from aqueous solutions. Chem. Eng. J..

[36] Yu R (2017). Graphene Oxide/Chitosan Aerogel Microspheres with Honeycomb-Cobweb and Radially Oriented Microchannel Structures for Broad-Spectrum and Rapid Adsorption of Water Contaminants. ACS Appl Mater Interfaces.

[37] Ho YS (2006). Second-order kinetic model for the sorption of cadmium onto tree fern: a comparison of linear and non-linear methods. Water Res..

[38] Repo E, Warchol JK, Bhatnagar A, Sillanpaa M (2011). Heavy metals adsorption by novel EDTA-modified chitosan-silica hybrid materials. J. Colloid Interface Sci..

[39] Plazinski W, Dziuba J, Rudzinski W (2013). Modeling of sorption kinetics: the pseudo-second order equation and the sorbate intraparticle diffusivity. Adsorption.

[40] Ngah WSW, Fatinathan S (2008). Adsorption of Cu(II) ions in aqueous solution using chitosan beads, chitosan-GLA beads and chitosan-alginate beads. Chem. Eng. J..

[41] Zeng L (2015). Chitosan/organic rectorite composite for the magnetic uptake of methylene blue and methyl orange. Carbohydr. Polym..

[42] Weber WJ, Morris JC (1963). Kinetics of adsorption on carbon from solution. J Sanit Eng Div.

[43] Wu FC, Tseng RL, Juang RS (2009). Initial behavior of intraparticle diffusion model used in the description of adsorption kinetics. Chem. Eng. J..

[44] Li Y (2014). Mechanical and dye adsorption properties of graphene oxide/chitosan composite fibers prepared by wet spinning. Carbohydr. Polym..

[45] Shi HC, Li WS, Zhong L, Xu CJ (2014). Methylene Blue Adsorption from Aqueous Solution by Magnetic Cellulose/Graphene Oxide Composite: Equilibrium, Kinetics, and Thermodynamics. Ind. Eng. Chem. Res..

[46] Ge HC, Chen H, Huang SY (2012). Microwave preparation and properties of O-crosslinked maleic acyl chitosan adsorbent for Pb2+ and (Cu2+). J. Appl. Polym. Sci..

[47] Ge H, Ma Z (2015). Microwave preparation of triethylenetetramine modified graphene oxide/chitosan composite for adsorption of Cr(VI). Carbohydr. Polym..

