# Supplementary material for: Hierarchical Porous Chitosan Sponges as Robust and Recyclable Adsorbents for Anionic Dye Adsorption
Source: Sci Rep. 2017 Dec 22;7:18054. doi: 10.1038/s41598-017-18302-0 (PMC5741733; doi:10.1038/s41598-017-18302-0)
Supplement: Supplementary file 2 — Supplementary Information [file 41598_2017_18302_MOESM2_ESM.pdf]

Supplementary Information

Hierarchical Porous Chitosan Sponges as Robust and Recyclable Adsorbents for Anionic Dye Adsorption

Mei Wang[a,b], Yifei Ma[a], Yan Sun[b], Sung Yong Hong[c], Stephanie K. Lee[b], Bumyong Yoon[c], Long Chen[d], Lijie Ci[d], Jae-Do Nam[b,c], Xuyuan Chen[a,e], Jonghwan Suhr[b,c,f*]

[a]State Key Laboratory of Quantum Optics and Quantum Optics Devices, Institute of Laser Spectroscopy, Collaborative Innovation Center of Extreme Optics, Shanxi University, Taiyuan, Shanxi 030006, China

[b]Department of Energy Science, Sungkyunkwan University, Suwon, 440-746, South Korea

[c]Department of Polymer Science and Engineering, Sungkyunkwan University, Suwon, 440-746, South Korea

[d]SDU & Rice Joint Center for Carbon Nanomaterials, Key Laboratory for Liquid-Solid Structural Evolution & Processing of Materials (Ministry of Education), School of Materials Science and Engineering, Shandong University, Jinan 250061, China

[e]Department of Micro- and Nanosystem Technology, Faculty of Technology and Maritime Sciences, University College of Southeast Norway, 3184 Borre, Norway

[f]School of Mechanical Engineering, Sungkyunkwan University, Suwon, 440-746, South Korea

*Corresponding Author: E-mail: suhr@skku.edu

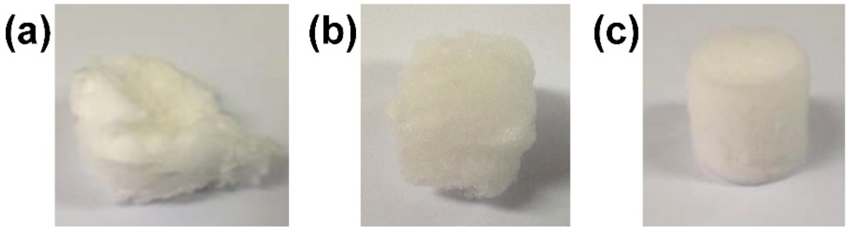

Figure S1. Digital images of Ch-5 foams derived from different freezing conditions: -196 °C (a), -80 °C (b), and -20 °C (c).

Table S1. Chemical associated groups corresponded to the characteristic peaks in the FT-IR spectra of chitosan foams.

**Wavenumber/cm^{-1}**	**Chemical associated groups**
897	ν(C-O-C) (symmetric stretching vibration)
1027	ν(C-O-C) (Saccharide structure-β-1,4)
1064	ν(C-O-C) (cyclic asymmetric stretching vibration)
1151	ν(C-O-C) (asymmetric stretching vibration)
1256	ν(C-N) Amine II
1316	ν(C-N) Amine I
1380	δ(CH2-OH)
1405	δ(CH-OH)
1545	δ(N-H) Amide
1637	ν(C=O) Amide
2870	ν(C-H)
3351	ν(O-H/N-H)

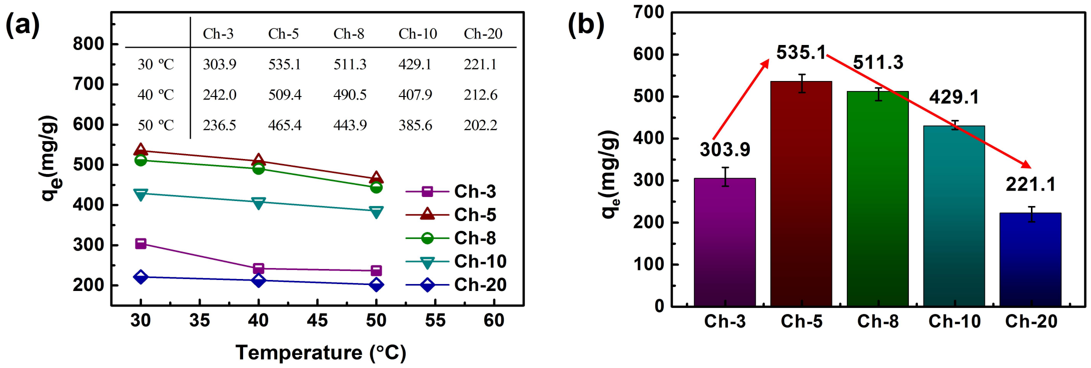

Figure S2. (a), The q_e values of different chitosan sponges under different temperatures. (b), The q_e values of chitosan sponges derived from different chitosan concentrations under 30 °C.

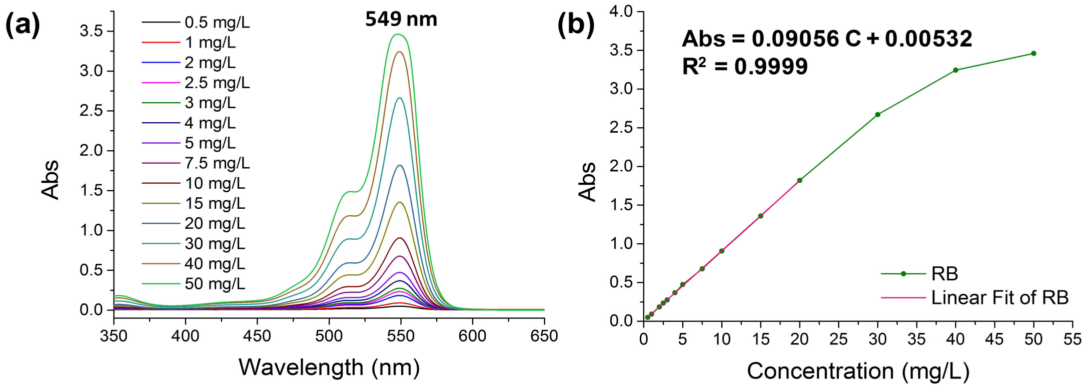

Figure S3. (a), UV-vis absorption spectra of RB solutions at different RB concentrations. (b), Linear relation of Abs and RB concentration.

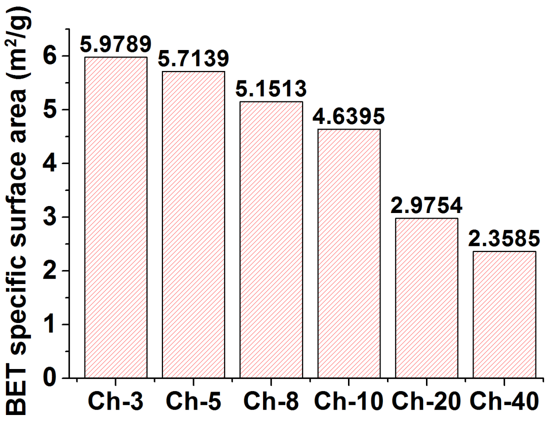

Figure S4. The BET specific surface areas of chitosan foams derived from the different concentration of chitosan solutions.

Table S2. Parameters of pseudo-first-order model for the adsorption of RB onto Ch-5 sponges.

C_0 (mg/L)	5	10	20	50	70	100	150	200
q_e (simulated)	462.3	539.8	387.9	358.8	392.7	599.9	426.8	405.8
k_1 (1/min)	0.0255	0.0238	0.0209	0.0164	0.0194	0.0266	0.0211	0.0220
R^2	0.9665	0.9546	0.9902	0.9708	0.9850	0.9301	0.9922	0.9851

Table S3. Parameters of pseudo-second-order model for the adsorption of RB onto Ch-5 sponges.

C_0 (mg/L)	5	10	20	50	70	100	150	200
q_e (simulated)	387.6	480.8	487.8	523.6	549.5	591.7	613.5	645.2
k_2 (g/mg min)	4.5E-5	3.8E-5	6.1E-5	6.3E-5	7.1E-5	7.2E-5	8.1E-5	9.1E-5
R^2	0.9930	0.9954	0.9978	0.9992	0.9996	0.9995	0.9996	0.9994

Table S4. Parameters of intra-particle diffusion model for the adsorption of RB onto Ch-5 sponges.

C_0 (mg/L)	5	10	20	50	70	100	150	200
C_1 (mg/g)	-44.4	-66.3	-58.5	-64.4	-43.3	-33.4	7.7	49.6
k_{1d} (g/mg $min^{1/2}$)	31.1121	43.2057	50.9741	59.3531	60.3043	64.6906	63.4070	61.6139
R^2	0.9982	0.9934	0.9966	0.9951	0.9968	0.9772	0.9487	0.9362
C_2 (mg/g)	31.1	44.4	143.7	232.2	279.2	282.1	270.3	309.4
k_{2d} (g/mg $min^{1/2}$)	22.1765	27.0146	22.7573	16.6690	15.6973	19.3196	23.6184	24.2986
R^2	0.9692	0.9923	0.9942	0.9901	0.9956	0.9876	0.9925	0.9505
C_3 (mg/g)	263.6	325.2	365.3	315.3	400.6	484.9	503.4	550.2
k_{3d} (g/mg $min^{1/2}$)	2.6816	3.7972	3.6916	9.7626	6.1636	3.1955	3.9592	3.1820
R^2	0.7212	0.9597	0.9594	0.9873	0.8115	0.9371	0.9968	0.9231

Table S5. Thermodynamic parameters (Gibbs free energy, enthalpy, and entropy changes) during the RB adsorption process for chitosan sponges with different densities at 30, 40 and 50 °C.

	Ch-3	Ch-5	Ch-8	Ch-10	Ch-20
ΔH (kJ/mol)	-16.95	-16.15	-17.74	-23.56	-53.21
ΔS (J/mol K)	-42.27	-29.36	-34.07	-49.54	-141.34
ΔG-30 (kJ/mol)	-4.14	-7.25	-7.42	-8.54	-10.34
ΔG-40 (kJ/mol)	-3.72	-6.96	-7.08	-8.05	-8.97
ΔG-50 (kJ/mol)	-3.30	-6.66	-6.74	-7.55	-7.56

Table S6. Comparison of the adsorption performances of chitosan sponges with other reported adsorbents for anionic dyes adsorption.

Anionic Dyes	Adsorbent	Adsorption capability (mg/g)	Recyclability (Y/N)	Adsorbate concentration (mg/L)	Reference
RB	**Chitosan** sponge	601.5	Y	200	**This work**
RB, methyl orange (MO)	Graphene-based aerogel	160, 250	Y	1 mM	J. Mater. Chem. A, 2013, 1, 7612-7621.
RB, Methyl Blue	Fe_3O_4@PDA/PEI	140, 170	Y	-	J. Mater. Chem. A, 2015, 3, 19960–19968.
RB	Poly(ether amine) @poly(vinylidene fluoride) (hPEA@PVDF) membranes	~ 560 umol/g (~ 545.3)	Y	300 μM	J. Mater. Chem. A, 2017, 5, 10470–10479
RB	Carbon nanotubes /magnetite/chitin nanocomposite	< 7	Y	5	Journal of Industrial and Engineering Chemistry, 2014, 20(5), 3559-3567.
RB, Calcein, MO	GO/sodium alginate/polyacrylamide ternary hydrogel	6.23, 6.23, 1.25	N	-	J. Mater. Chem. A, 2013, 1, 7433-7443.
RB	Amberlite Ira-938 resin	325	N	50	Desalination and Water Treatment, 2016, 57, 13527–13533.
RB	Bottom ash	< 5	N	0.02 mM-0.1mM	RSC Advances, 2012, 2, 8381-8389.
Congo red (CR)	GO/polyacrylamide composite hydrogels	13	Y	70	J. Mater. Chem. A, 2015, 3, 17445-17458.
CR	**Chitosan**/montmorillonite	53.42	N	100-225	Journal of Hazardous Materials, 2007, 147, 979–985.
CR	Dual-porosity Mn_2O_3 cubes	125.6	Y	-	Journal of Hazardous Materials, 2017, 333, 222–231
CR, Orange G	PPy-derived carbon nanotube framework	151,122	N	40	Applied Surface Science, 2017, 414, 218–223.
MO	**Chitosan**/graphene oxide composite	0.8 mmol/g (261.9)	Y	1.6 mM	Chemical Engineering Journal, 2016, 284, 1397–1405.
MO	**Chitosan**/organic rectorite composite	5.56 (Simulated)	Y	10-160	Carbohydrate Polymers, 2015, 123, 89-98.
MO	Core−Shell Structured Graphene-**Chitosan** Beads	353	N	210	ACS Appl. Mater. Interfaces, 2015, 7, 14439−14445.

MO, Eosin Y	GO/**chitosan** aerogel microsphere	554.7,419.8	N	500	ACS Appl. Mater. Interfaces, 2017, 9, 21809−21819.
MO	Poly-dopamine (PD) /GO composites	30	N	-	J. Mater. Chem. A, 2014, 2, 5034–5040
Alizarin yellow R	Magnetic **chitosan** with GO	1.49-14.82	Y	20-260	Carbohydrate Polymers, 2016, 152, 520-531.
Eosin Y	GO-**chitosan** hydrogels	326	N	100	J. Mater. Chem. A, 2013, 1, 1992–2001.
Reactive Blue 19	**Chitosan**/oil palm	909.1 (Simulated)	N	50-500	Chemical Engineering Journal, 2008, 136, 164–172
Acid violet	**Chitosan**/ polyurethane	29.6 (Simulated)	N	-	Fibers and Polymers, 2009, 10(5), 636-642.
Reactive dye RR222	**Chitosan**/activated clay	1912 (Simulated)	N	-	Journal of Colloid and Interface Science, 2004, 278, 18–25.
Tartrazine	**Chitosan**/bentonite	294.1 (Simulated)	N	300	Water Air Soil Pollut, 2010, 206, 225–236.
Acid fuchsin	CNT-graphene hybrid aerogel	35.8	N	20	J. Mater. Chem., 2012, 22, 8767-8771.
Metanil yellow	Polyaniline-bentonite composite	444.44	N	100-600	Colloid Polym Sci.,2017, 295, 1165–1175.
Sunset Yellow	PEI/nylon microfiltration membrane	600	Y	25 ppm	Journal of Hazardous Materials, 2017, 337, 217–225
